# The utility of point of care serum lactate in predicting serious adverse outcomes among critically ill adult patients at urban emergency departments of tertiary hospitals in Tanzania

**DOI:** 10.1186/s41182-019-0186-1

**Published:** 2019-12-27

**Authors:** Uwezo Edward, Hendry R. Sawe, Juma A. Mfinanga, Theresia A. Ottaru, Michael Kiremeji, Deus N. Kitapondya, Dereck A. Kaale, Asha Iyullu, Nicks Bret, Ellen J. Weber

**Affiliations:** 1 Emergency Medicine Department, Muhimbili University of Health and Allied Science, P.O. Box 65001, Dar es Salaam, Tanzania; 2 Emergency Medicine Department, Muhimbili National Hospital, Dar es Salaam, Tanzania; 3 Muhimbili University of Health and Allied Science, Dar es Salaam, Tanzania; 4 Department of Emergency Medicine, Wake Forest School of Medicine, Winston-Salem, NC USA; 5 Department of Emergency Medicine, University of California, San Francisco, CA USA

**Keywords:** Lactate level, Serious adverse outcomes, Emergency medicine, Emergency care, Tanzania

## Abstract

**Background:**

Elevated serum lactate levels have been shown in numerous studies to be associated with serious adverse events, including mortality. Point of care lactate level is increasingly available in resource-limited emergency department (ED) settings. However, little is known about the predictive ability of for serious adverse events.

**Aim of the study:**

We aimed to describe the utility of serum lactate level as a predictor of 24-h serious adverse events among adult patients presenting at the Emergency Medicine Department (EMD) of Muhimbili National Hospital (MNH) and MUHAS Academic Medical Center (MAMC).

**Methods:**

This was a prospective observational study involving adult patients presenting to the EMD-MNH and MAMC from 1 September 2018 and 31 October 2018. Eligible patients with at least one lactate level test drawn while in the ED were examined in terms of their demographics, relevant clinical characteristics, and any serious adverse event (SAE) within 24 h of arrival. The sensitivity and specificity of lactate level to predict outcomes of interest were determined using the best cut-off point constructed from AUROC to see how well lactate level could discriminate which patients would have adverse events in the next 24 h. Categorical and continuous variables were compared with the chi-square test and two-sample *t* test, respectively.

**Results:**

We screened 2057 (20.9%) out of 9828 patients who presented during study period, and enrolled 387 (18.8%). The overall median age was 54 years (interquartile range 40–68 years) and 206 (53.2%) were male. Using local triaging system, a total of 322 (83.2%) was triaged as an emergency category. The mean lactate level was 3.2 ± 3.6 mmol/L, 65 (16.8%) patients developed at least one SAE, with 42 (11%) who required ICU/HDU, 37 (10%) needed ventilator support, 10 (3%) required inotropes, and 9 (2%) developed cardiac arrest. The overall 24-h mortality was 28 (7%). The AUC of serum lactate level for overall 24-h mortality was 0.801 (95%CI, 0.7–0.9, *P* ≤ 0.001). At the optimal cutoff value (3.8 mmol/L), lactate level had a sensitivity and specificity for 24-h mortality of 64% and 85%, respectively. Mortality of the high-lactate level group (^3^3.8 mmol/L) was significantly higher than that of the low-lactate level group (< 3.8 mmol/L), 23.8% vs. 2.9%, respectively (95%CI 3.8–17.2, *p* < 0.001), with the relative risk of mortality in the high-lactate level group being 8.1 times higher compared to the low-lactate level group.

**Conclusion:**

The utility of lactate level in predicting mortality was similar to that seen in high-resource settings. A serum lactate level of ^3^3.8 mmol/L predicted 24-h serious adverse events in unselected patients seen in the high-acuity area of our ED. Incorporating serum lactate level in ED in lower- and middle-income countries (LMICs) can help identify patients at risk of developing serious adverse events.

## Background

High-lactate level has been linked to an increased risk of overall mortality in a wide range of populations, including adults and the elderly, regardless of whether they have infections or not [1, 2]. Elevated lactate level is a marker of poor organ perfusion and has shown to be useful as a marker of poor prognosis in clinically ill patients [3–5]. Most of the literature-based in clinical settings has focused on sepsis and septic shock [6–10]. However, there are many causes of elevated lactate level besides those related to sepsis, which include cardiac arrest, trauma, seizures, ischemia, diabetic ketoacidosis, thiamine deficiency, malignancy, liver dysfunction, genetic disorders, toxins, medications, or any form of shock [11–14]. Recent literature suggests a lactate level less than 2 mmol/l as normal whilst lactate level above 4 mmol/l has an association with increased mortality and longer hospital stay [2, 5, 10].

The availability and utilization of lactate level testing, especially blood gas point-of-care (POC) testing, has increased worldwide, especially in emergency settings [10]. Despite the wide use of POC lactate level in the developed world and its promise in improving patient care [10, 14–16], there is a paucity of African-based studies on the utility of this biomarker, a few of which include (1) serum lactate level as a biomarker for intestinal ischemia done in Uganda [15], (2) point-of-care lactate level testing predicts mortality of severe sepsis in a predominantly HIV type 1 in Uganda [16], and (3) POC lactate level analysis in obstetric sick patients done in 2016 in Malawi [17]. In Tanzania, one study was done at Kilimanjaro Catholic Medical Center (KCMC) which assessed blood lactate level in children presenting to the hospital with febrile illnesses [18]. Most of these studies were done on selected diseases and populations. The “surviving sepsis campaign” integrated lactate level into “early goal-directed therapy” as a marker of adequate resuscitation and monitoring [8]. Some centers have included lactate level as a triaging tool for risk stratification and a predictor of poor outcomes in the ED population [19, 20]. Early illness recognition and early resuscitation with a focus on early lactate level clearance have been shown to improve outcomes in sepsis [10, 19, 21]. Although, it is not known if lactate level is useful in resource-limited settings, where there is a broad, undiagnosed, and untreated disease burden presenting in an undifferentiated manner.

We intended to investigate the utility of POC lactate level and its capacity for predicting serious adverse outcomes in critically ill adult patients presenting to the emergency department and triaged to the resuscitation rooms regardless of the underlying disease conditions.

## Methods

### Study design

This was a prospective, observational study of a convenience sample of adult (> 18 years of age) patients presenting to the Emergency Medicine Department of Muhimbili National Hospital and MUHAS Academy Medical Centers who had at least one POC lactate level result within 1 h of arrival to the Emergency Medicine Department during the period of study. Ethical approval was obtained from the Institution review board of MUHAS (IRB) and Muhimbili National Hospital (MNH) ethical boards.

### Study setting

This study was conducted in the emergency departments of two hospitals in Dar-es-Salaam, Tanzania: Muhimbili National Hospital (MNH) and MUHAS Academic Medical Center (MAMC). MNH is located in the Ilala district, along the United Nations Road in Upanga. It has a bed capacity of 1500 and serves as a referral hospital for the whole of Tanzania. The ED was established in 2010 through a partnership between the Ministry of Health and Social Welfare and the Abbott Fund Tanzania. The ED is the first full capacity public ED in Tanzania and the site for the emergency medicine (EM) residency training program. The department is staffed 24 h, 7 days a week by trained emergency medicine specialists, who oversee the care of patients and supervise interns, registrars, and EM residents. The ED sees an average of 45,000 patients annually with an admission rate of 65% [22].

MAMC is a recently inaugurated full capacity health facility occupying 3800 acres, located 3 km off the Dar-es-Salaam-Morogoro highway, approximately 31.2 km from MNH. The state-of-the-art emergency medicine department is operating under the direction of an emergency medicine specialist faculty from MUHAS 24-h a day who provides clinical supervision on patients care given by intern doctors, registrars, and emergency medicine residents. Nearly all admissions to these two hospitals come through the emergency medicine departments as the main entry point.

### Study protocol

The study inclusion criteria were adults, aged 18 years or older, who were triaged into resuscitation rooms using the Emergency Severity Index (ESI), with initial serum lactate levels obtained while in the ED, both at MAMC and MUHAS within the first hour of arrival. Written informed consent was provided and signed on approval to all screened patients before enrolment. Venous blood samples were drawn at the discretion of the treating physician as a standardized practice in the ED. At both facilities, lactate level was evaluated using a point of care test (i-STAT®1 analyzer MN: 300-G manufactured by Flextronics manufacturing Singapore Pte Ltd., for Abbott Point of Care Inc. USA 2013). This portable clinical analyzer device uses the i-STAT lactate level cartridges and requires approximately 2.5 mL of blood in a heparinized syringe. The blood was drawn by clinicians in the ED and analyzed by a trained technician: 2 to 3 drops of blood was inserted into the cartridge; then, the cartridge was inserted into the analyzer, and results were available in approximately 2 min. Test results were uploaded manually by nurses or the attending clinician in the WellSoft electronic medical record system used at the emergency medicine departments. Admitted patients were followed up in their respective disposition ward manually within 24 h and those who were discharged from the ED received a phone call after 24 h for outcomes by the research assistants. We defined serious adverse outcomes as the need for ICU/HDU, vasopressor/inotropes, cardiac arrest, mechanical ventilator support, and overall mortality.

### Data analysis

Data was entered in online data Capture Software (RED-Cap version 7.2.2, Vanderbilt, Nashville, TN, USA) and the data was exported to Statistical Package for Social Science (SPSS version 23.0, IBM, Ltd., North Carolina, USA) for analysis. Categorical variables were summarized as frequencies and percentages, and continuous variables as means and standard deviations (SD) or medians and interquartile ranges (IQR), depending on data distribution. A receiver operating characteristics curve was constructed for 24-h mortality, and each serious adverse event and the lactate level best cutoff value was chosen as a maximum value on the Youden’s index formula [23]. Then, we dichotomized patients into high- and low-lactate level groups respectively based on the optimal cut-off point for 24-h mortality to determine the association between lactate level and clinical presentation, initial laboratory tests, ED management, disposition plan, and serious adverse outcomes. Categorical variables were compared with the chi-square test or Fisher’s exact test; two-tailed *p* values of < 0.05 were considered statistically significant. All the abnormal vital signs ranges were drawn from a previous study on the association between abnormal vital sign groups and undesirable patient outcomes [24]. Also, the abnormal ranges for blood tests were based on the specifications of our machines (i-STAT®1 analyzer MN: 300-G manufactured by Flextronics manufacturing Singapore Pte Ltd., for Abbott Point of Care Inc. USA 2013) and reference ranges were calibrated as per manufacturer instructions. ED admitting diagnosis from patients file were considered for analysis and disease categorization were done based on admitting diagnosis and chief complaints and was only if information’s are available at admission.

## Results

**Fig. 1:**
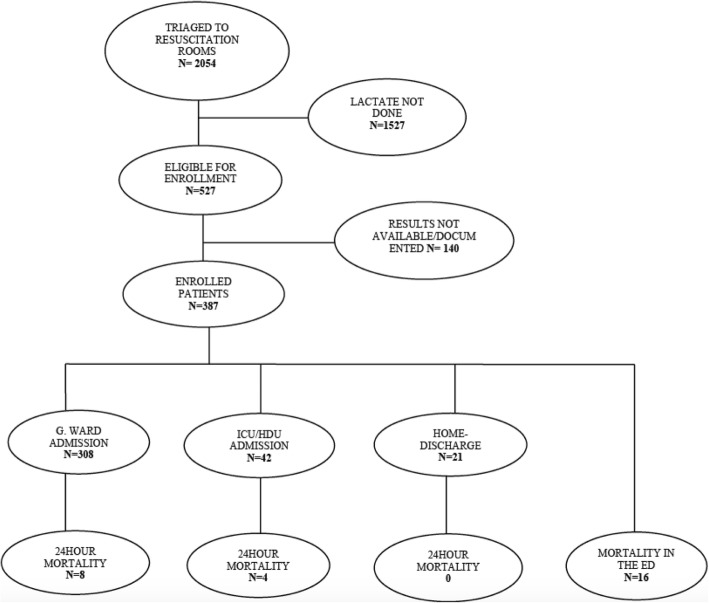
Study flow diagram

We screened 2054 (100%) patients triaged to resuscitation rooms as level I and II during the study period. Of these, 527 (25.7%) adult patients had lactate level obtained and they all consented to the study. Of those who consented, 387 (73.4%) had available lactate level results and were included in the final analysis (Fig. 1).

### Patient characteristics

The overall median age was 54 years (interquartile range 40–68 years) and 206 (53.2%) were male. Three hundred twenty-two (83.2%) triaged as an emergency category with 195 (50.4%) being referred from peripheral hospitals and less than half, and 174 (45%), with insurance coverage. Diabetes mellitus, HIV, and malignancy were the leading comorbidities, 42 (10.8%), 24 (6.2%) and 15 (3.9%), respectively (Table 1).

**Table 1:** Patients demographics characteristics

Variables	*N* = 387 *n* (%)
Median Age (IQR)	54 (40–68)
Sex	
Male	206 (53.2)
Age (years)	
< 50	165 (42.6)
> 50	222 (57.4)
Referral status	
Self-referred	195 (50.4)
Triage levels	
Emergency	322 (83.2)
Priority	65 (16.8)
Payment status	
Insured	174 (45.0)
Cash payment	213 (55.0)
Education profile	
Formal education	340 (87.9)
Informal education	47 (12.1)
Comorbidities	
Diabetes mellitus	42 (10.8)
HIV	24 (6.2)
Malignancy	15 (3.9)
Chronic renal insufficiency	14 (3.6)
Hypertension	9 (2.3)

Complaints of general body malaise and abdominal symptoms were the most common chief complaints involving 54 (13.9% for each), followed by difficulty in breathing and altered mental status. Tachycardia (193, 49.9%) and tachypnea (155, 40.1%) were the most frequent abnormal vital signs. Heart failure (46, 11.9%), malignancies (43, 11.1%), and cerebrovascular accident (42, 10.8%) were the leading ED diagnoses. However, infectious (71, 18.3%) and cardiovascular (68, 17.8%) represented the main disease categories (Table 2).

**Table 2:** Patients baseline variables and clinical characteristics

Variables	*N* = 387 *n* (%)
Presenting complaints	
General body malaise	54 (13.9)
Abdominal symptoms	54 (13.9)
Difficulty in breathing	30 (7.7)
Altered mental status	30 (7.7)
Cough	28 (7.2)
Initial vitals	
Tachycardia (HR > 100beat/min)	193 (49.9)
Tachypnoea (RR > 20breath/min)	155 (40.1)
Altered mental status (GCS < 15)	91 (23.5)
Hypoxia (SPO2 < 90%)	56 (14.5)
Febrile (> 37.9C)	32 (8.3)
Hypotension (SBP < 90 mmHg)	29 (7.5)
Bradycardia (HR < 60beat/min)	10 (2.6)
ED diagnosis	
Heart failure	46 (11.9)
Malignancies	43 (11.1)
Cerebrovascular disease	42 (10.8)
Main disease categories	
Infectious	71 (18.3)
Cardiovascular	68 (17.8)
Malignancy	46 (11.9)
Cerebrovascular	42 (10.9)

### EMD laboratory investigations and management plan

Among the 387 enrolled patients with serum lactate level, the mean lactate level was 3.2 ± 3.6 mmol/L. Two hundred five (53%) had elevated level ≥ 2 mmol/l, with 133 (34.4%) of the total samples having lactate level between 2 to < 4 mmol/L and 72 (18.6%) of the total having lactate level ^3^4 mmol/L. Additional testing was at the discretion of the physician. Of the 380 patients that had sodium tested, 209 (55%) had hyponatremia, 174 (51.8%) out of 336 tested had elevated BUN, and creatinine was elevated in 153 (47.7%) out of 321 patients tested. Approximately half of those tested had an abnormal WBC. In the ED, most of the enrolled patients were given intravenous fluids (328, 84.8%). Although only 18% of the sample were suspected to have an infectious etiology, 42.9% received antibiotics. Oxygen therapy was provided to 26.9% of the patients. Invasive procedures were performed on 22 (5.7%) patients in the ED and 9 (2.3%) underwent cardiopulmonary resuscitation (Table 3).

**Table 3:** Patient’s EMD laboratory investigations and management plan

Variables	*n* (%)
Laboratory investigations	
Serum lactate mean 3.2 ± 3.6 mmol/L	
Lactate levels (mmol/L) N = 387	
< 2	182 (47.0)
2 to < 4	133 (34.4)
≥ 4	72 (18.6)
Hyponatremia (Na^+^ < 136 mmol/L), *N* = 380	209 (55)
High urea (Serum Ur > 6 mmol/L), *N* = 336	174 (51.8)
High creatinine (serum Cr > 115 μmol/L), *N* = 321	153 (47.7)
WBC^Ω^ < 4 or > 11, *N* = 150	71 (47.3)
PH (acidemia < 7.35), *N* = 287	100 (34.8)
Hypokalemia (K^+^ < 3.5 mmol/L), N = 379	112 (29.6)
Hemoglobin (< 7 g/dl), *N* = 266	41 (15.4)
mRDT positive, *N* = 371	24 (13.7)
Hyperkalemia (K^+^ > 5.5 mmol/L), *N* = 379	48 (12.7)
Low RBG, *N* = 371	19 (5.1)
HIV positive, *N* = 332	13 (3.9)
EMD management (*N* = 387)	
Intravenous fluids	328 (84.8)
Antibiotics	166 (42.9)
Oxygen therapy	104 (26.9)
Intubations	22 (5.7)
Blood transfusion	21 (5.4)
Inotropes/vasopressors	10 (2.6)
Cardiopulmonary resuscitation	9 (2.3)

^Ω^Abnormal WBC in cells per cubic millimeter

### Determining the optimal cut-off value of lactate level to predict adverse events

A series of receiver operating characteristic curves were plotted to determine the area under the curve (AUROC), and the best cut-off point of lactate level for the following outcomes of interest, for 24-h mortality, need for inotropes/vasopressor, cardiac arrest, need for mechanical ventilator, and overall serious adverse events (SAEs).

### Twenty-four-hour mortality

**Fig. 2:**
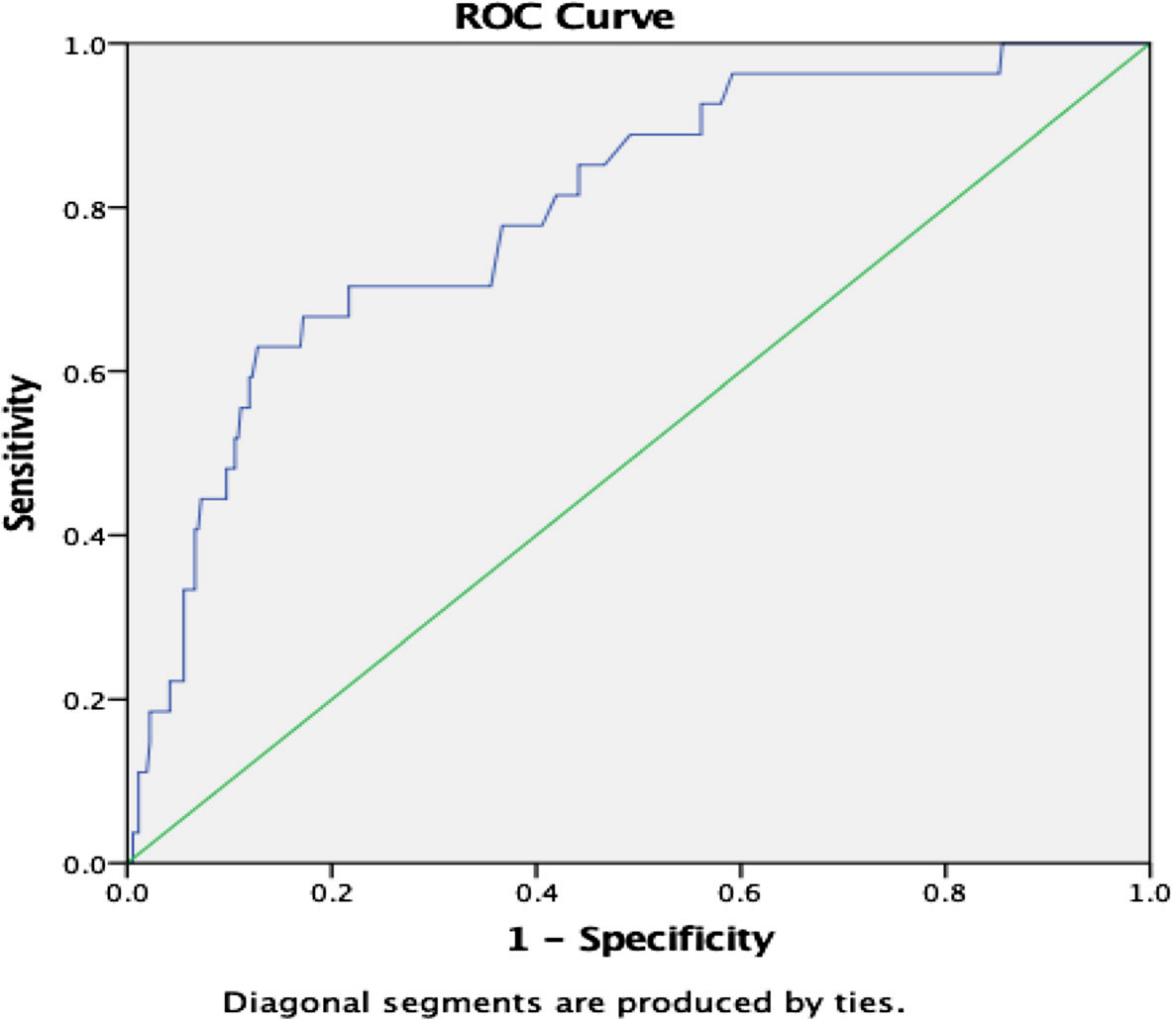
Graphic representation of ROC curve for 24-h mortality

The lactate level had an AUROC of 0.80 for 24-h mortality (95% CI, 0.7–0.9, *p* < 0.0001). The optimum cut-off value using Youden’s Index was >/= 3.8mmol/L with sensitivity and specificity of 64% and 85%, respectively (Fig. 2).

### Need for inotrope/vasopressor

**Fig. 3:**
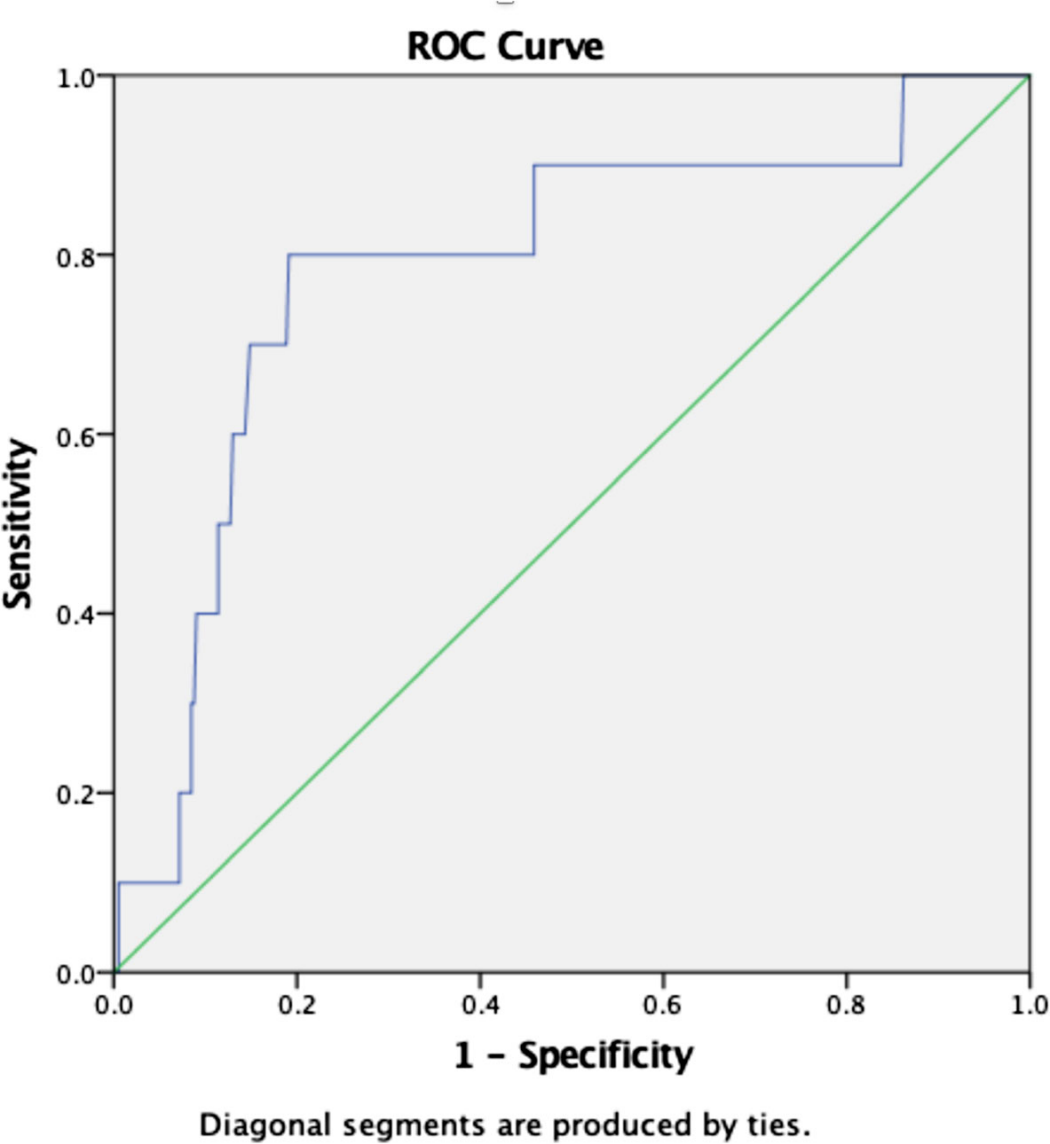
Graphic representation of ROC curve for need for inotrope/vasopressors

The AUROC for lactate level in predicting the need for inotropes or vasopressors was 0.79 (95%CI, 0.6–0.9, *p* < .002). The optimum cut-off value was 3.6 mmol/L with sensitivity and specificity of 80% and 80%, respectively (Fig. 3).

### Cardiac arrest

**Fig. 4:**
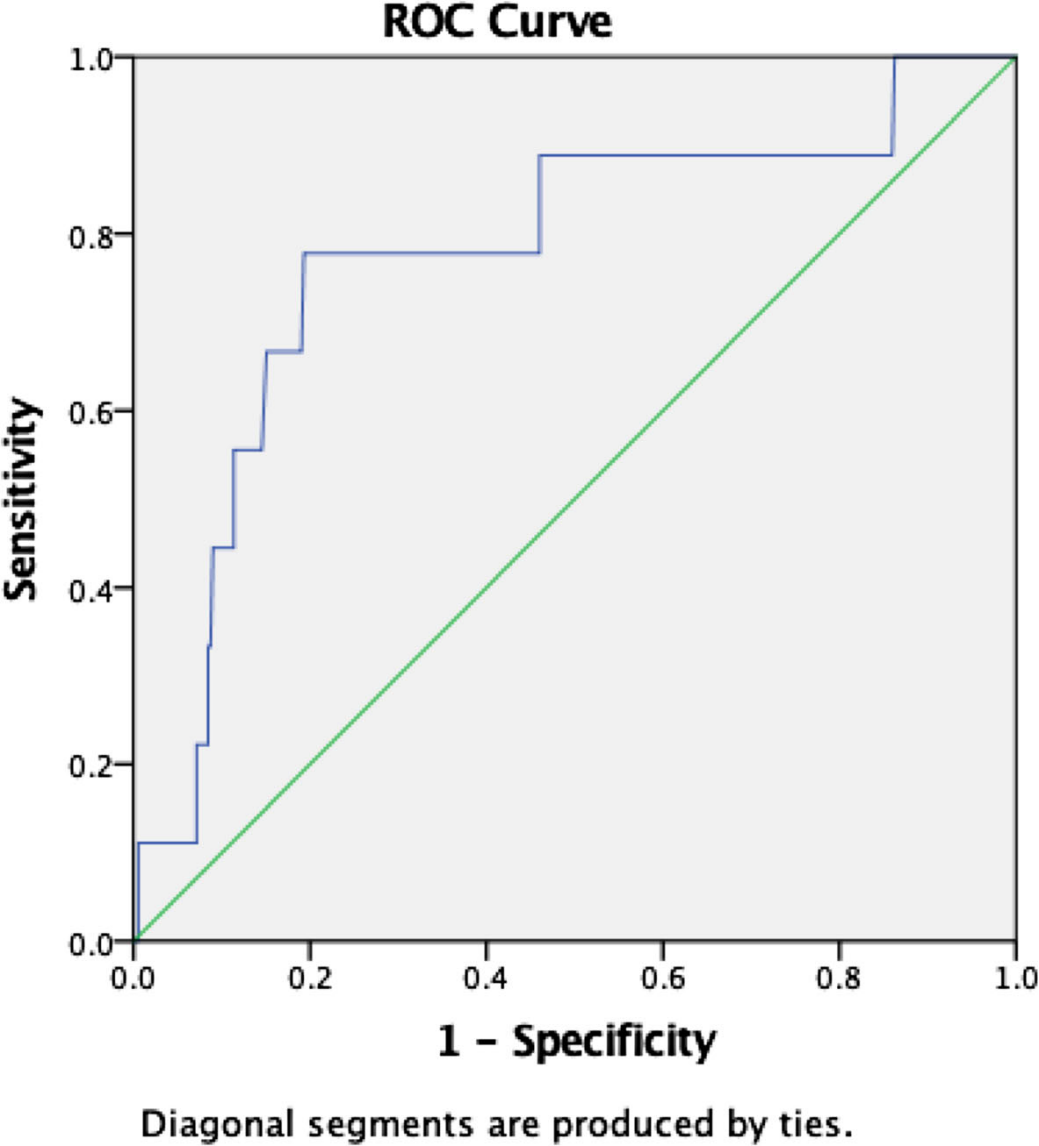
Graphic representation of ROC curves for cardiac arrest

Lactate level predicted cardiac arrest with an AUROC of 0.78 (95%CI, 0.6–0.9, *p* = 0.005). The optimum cut-off value was 3.5 mmol/L, with sensitivity and specificity of 78% and 80%, respectively (Fig. 4).

### Need for mechanical ventilator support

**Fig. 5:**
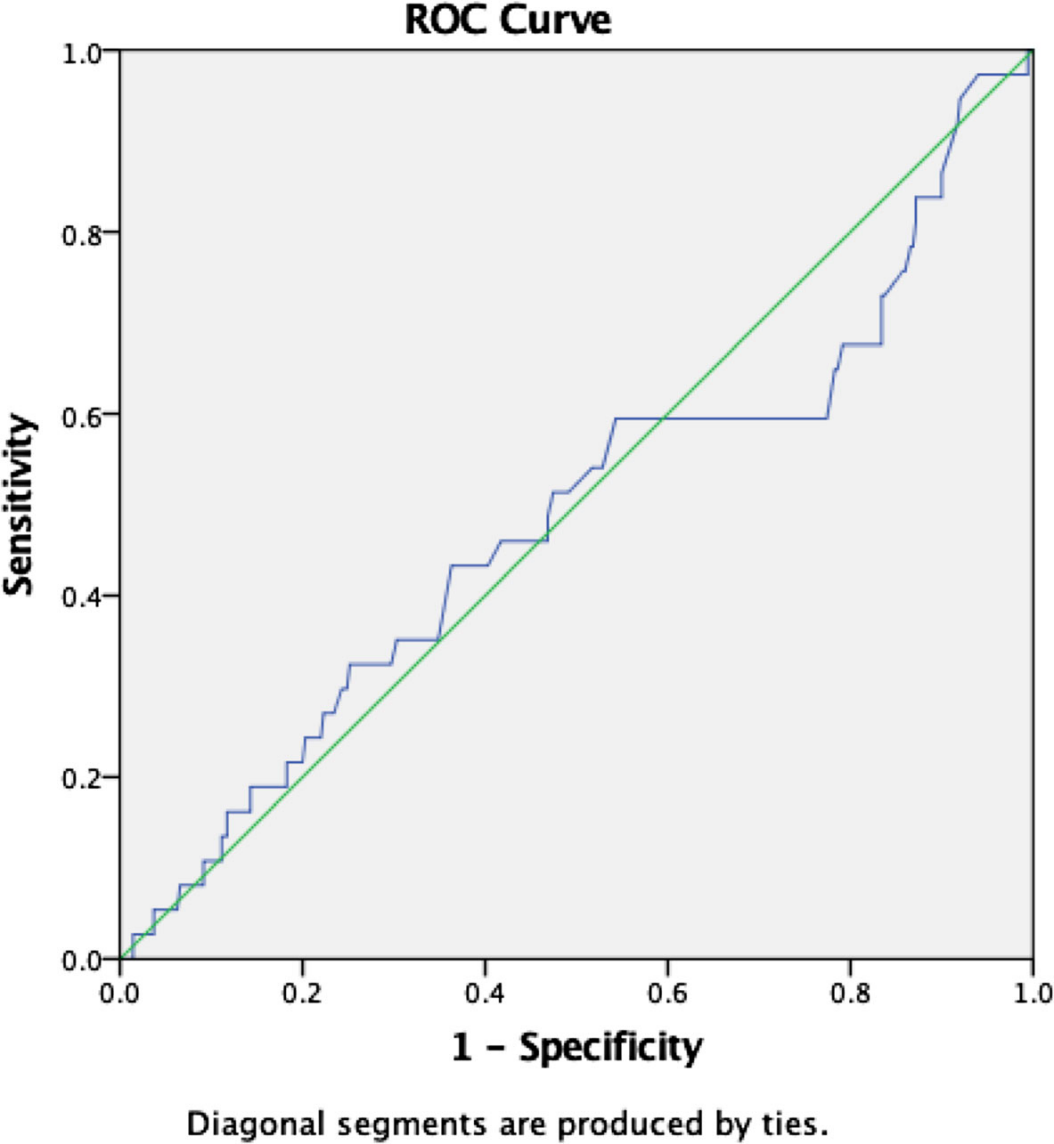
Graphic representation of ROC curves needs for mechanical ventilator

The AUROC of lactate level for prediction of mechanical ventilator support was 0.49 (95%CI, 0.4–0.6, *p* = 0.790), essentially indicating no discrimination. The optimum cut-off value was 3.4 mmol/L with sensitivity and specificity of 24% and 78%, respectively (Fig. 5).

### Overall serious adverse events (SAEs)

**Fig. 6:**
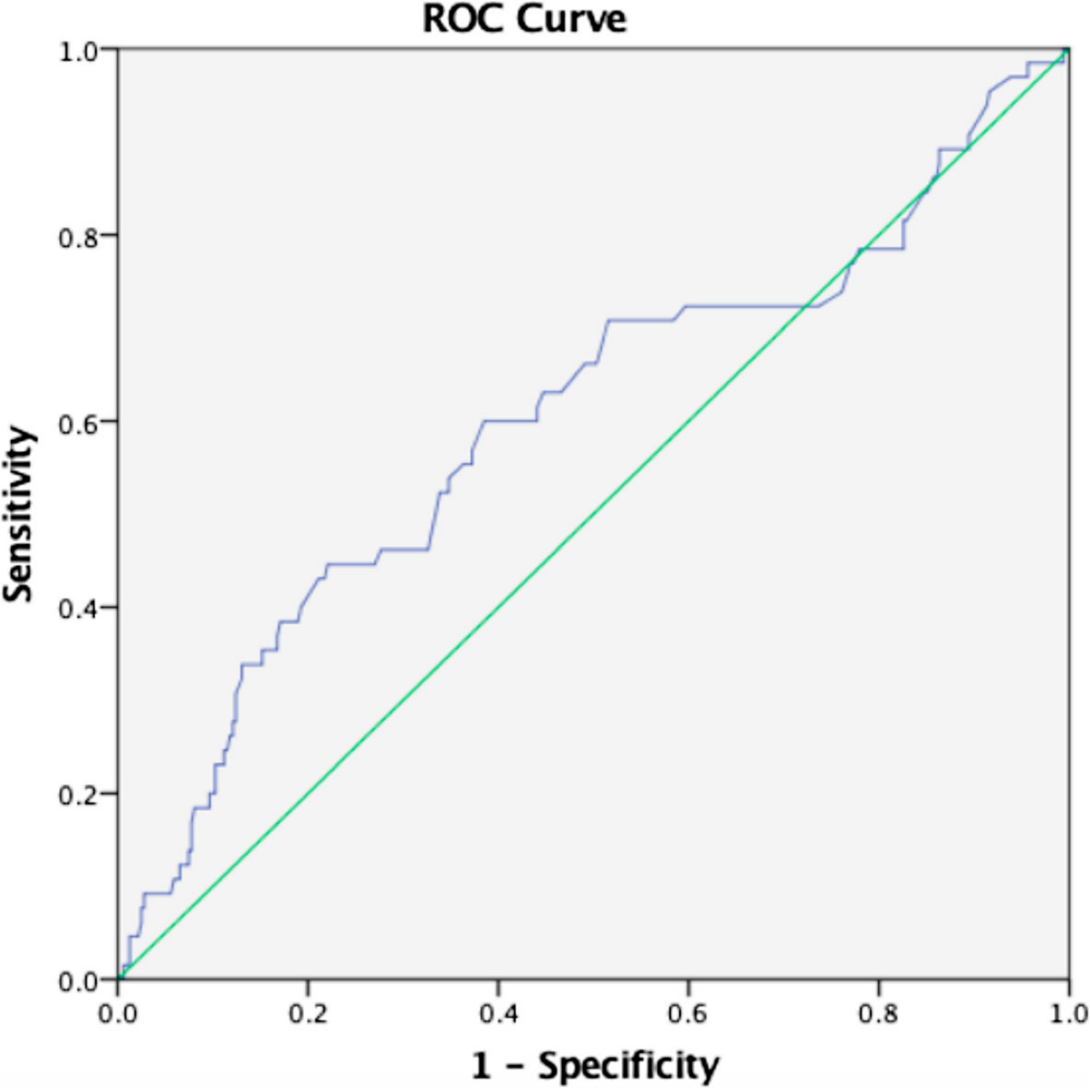
Graphic representation of ROC curves for overall SAEs

For any serious adverse event, including mortality, need for vasopressor/inotropes, mechanical ventilation, and cardiac arrest, the AUROC was 0.60 (95%CI, 0.5–0.7, *p* = .009). The optimum cut-off value was 3.5 mmol/L with sensitivity and specificity of 40% and 80%, respectively (Fig. 6).

### Baseline variables, laboratory investigation, and management at ED with stratified lactate level

We used the lactate level associated with 24-h mortality to dichotomize patients into high vs. low lactate level (≥ 3.8 mmol/L and low < 3.8 mmol/L) and determine the association of a high vs. low lactate level with clinical presentation, laboratory investigations performed in the ED, and management at ED. In this study, we found hypotension, tachycardia, tachypnea, and hypoxia all had a significant association with a high lactate level.

Both hyperkalemia and hypokalemia were found to have a statistically significant association with the high lactate level. Lactate level was also significantly associated with low PH level (acidemia), abnormal WBC (< 4, > 11) and elevated serum urea.

Most of the patients in our study received intravenous fluid (328, 84.8%), antibiotics (166, 42.9%) and oxygen therapy (104, 26.9%) with no statistically significant association to lactate level. However, intubation (*p* = 0.016) and inotropes/vasopressors (*p* < 0.001) were significantly associated with high lactate level (Table 4).

**Table 4:** Baseline variables, investigations and management by lactate level

	Overall *N* = 387 (*n*)	Serum lactate (mmol/L)		*P* value
		< 3.8, *N* = 307 *n* (%)	≥ 3.8, *N* = 80 *n* (%)	
Initial characteristics				
Hypotension (SBP < 90 mmHg)	29	13 (4.2)	16 (20.0)	< 0.001
Tachycardia (HR > 100 beat/min)	193	142 (46.3)	51 (63.7)	0.005
Bradycardia (HR < 60 beat/min)	10	7 (2.3)	3 (3.8)	0.438
Tachypnoea (RR > 20 breath/min)	316	242 (78.8)	74 (92.5)	0.005
Hypoxia (^Φ^SPO2 < 90%)	56	35 (11.4)	21 (26.3)	0.001
Febrile (^ϖ^Temp > 37.9 °C)	32	24 (7.8)	8 (10.0)	0.528
Altered mental status (GCS < 15)	91	69 (22.5)	22 (27.5)	0.345
Age (years)				
< 50	165	120 (39.1)	45 (56.3)	0.006
> 50	222	187 (60.9)	35 (43.8)	
Laboratory tests				
High (serum Cr-153 μmol/L)	153	117 (38.1)	36 (45.0)	0.262
High serum Urea(> 9.7 mmol/L)	174	127 (41.4)	47 (58.8)	0.005
Hyperkalemia (K^+^ > 5.5 mmol/L)	338	274 (89.3)	64 (80.0)	0.027
Hypokalemia (K^+^ < 3.5 mmol/L)	112	97 (31.6)	15 (18.8)	0.024
Hyponatremia (Na^+^< 136 mmol/L)	209	162 (52.8)	47 (58.8)	0.339
WBC^a^	77	71 (23.1)	6 (7.5)	0.002
Malaria test positive	24	17 (10.1)	7 (15.9)	0.290
Hemoglobin (< 7 g/dL)	41	34 (11.1)	7 (8.8)	0.547
Low RBG (< 3 mmol/L)	9	5 (1.6)	4 (5.0)	0.092
HIV rapid test positive	13	10 (23.6)	3 (27.3)	0.709
Acidemia (serum-PH < 7.35)	100	90 (29.3)	10 (12.5)	0.002
EMD management				
Oxygen therapy	104	87 (28.3)	17 (21.3)	0.203
Intravenous fluids	328	257 (83.7)	71 (88.8)	0.264
Blood transfusion	21	15 (4.9)	6 (7.5)	0.358
Antibiotics	166	130 (42.3)	36 (45.0)	0.669
Inotropes/vasopressor	10	2 (0.7)	8 (10.0)	< 0.001
Intubations	22	13 (4.2)	9 (11.3)	0.016
Cardiopulmonary resuscitation	9	2 (0.7)	7 (8.8)	< 0.001

^Ω^Abnormal WBC in cells per cubic millimeter

^Φ^Saturation of oxygen in peripheral capillary

^ϖ^Measurements were all axillary

*AMS* altered mental status from patients’ baseline. Blood transfusion included whole blood and packed RBCS, intravenous fluid-crystalloids

### Patient disposition and hospital outcomes

Those patients in the high-lactate level group were admitted to the general ward (58, 72.5%), and the ICU (10, 12.5%); 11 (13.8%) died in the ED and one patient (1.3%) was discharged home. The mortality rate among those with high lactate level admitted to general ward (4, 50%) was the same as those with low lactate (50%) in the low-lactate level group; the mortality was 100% in ICU for those in the high-lactate level group while there were no deaths in the ICU for patients with lactate level < 3.8 mmol/L with *p* = 0.002. The mortality rate in ED among those with a lactate level ≥ 3.8 was 11 (13.8%), compared to 5 (1.6%) for those with lactate level below the cut-off (relative risk 8.4; 95%CI, 3.0–24, *p* = < 0.001). The overall mortality at 24 h was 23.8% vs. 2.9% in high- vs. low-lactate level group respectively, with the relative risk of dying within 24 h being 8.1× higher in the high-lactate level group compared to those with lactate level < 3.8 mmol/L (Table 5).

**Table 5:** Patients disposition and hospital outcomes

Variables	Overall *N* = 387 *n* (%)	Serum lactate (mmol/L)		Relative risk (95% CI)	*P* value
		< 3.8 *N* = 307 *n* (%)	≥3.8 *N* = 80 *n* (%)		
General ward admission	308	250 (81.2)	58 (72.5)	0.9 (0.8–1.0)	0.077
ICU/HDU admission	42	32 (10.4)	10 (12.5)	1.2 (0.6–2.3)	0.595
Discharged home from ED	21	20 (6.5)	1 (1.3)	0.2 (0.0–1.4)	0.092
Death in the ED	16	5 (1.6)	11 (13.8)	8.4 (3.0–24)	< 0.001
Death in ICU/HDU	4	0 (0.0)	4 (5.0)	5.0 (4.1–6.2)	0.002
Death in General ward	8	4 (1.3)	4 (5.0)	3.8 (1.0–15)	0.061
24 h mortality	28	9 (2.9)	19 (23.8)	8.1 (3.8–17)	< 0.001

*CI* confidence interval

### Twenty-four-hour serious adverse events with the distribution of high vs. low lactate level (high < 3.8 mmol/L and low ^3^3.8 mmol/L)

The relative risk for 24 h Serious Adverse Events and high vs. low lactate level (high ≥ 3.8 mmol/L and low < 3.8 mmol/L) among our population of the study. The relative risk for requiring a vasopressor was 15.4 (95%CI, 3.3–70.) and 13.4 (95%CI, 2.9–63.4) for cardiac arrest among patients with high lactate level—noting the wide confidence intervals which may reflect the small number of patients for each outcome (Table 5). As predicted from the ROC curve regarding the need for mechanical ventilation and the need for ICU/HDU, there was no association with high lactate level meaning in this study elevated lactate level was not predictive for mechanical ventilation or ICU/HDU disposition (Table 6).

**Table 6:** 24-h serious adverse events with distribution of high vs. low lactate level (high ≥ 3.8 mmol/L and low < 3.8 mmol/L)

Variables	Overall *N* = 387 *n* (%)	Serum lactate (mmol/L)		Relative risk (95% CI)	*P* value
		< 3.8 *N* = 307 *n* (%)	≥ 3.8 *N* = 80 *n* (%)		
Need for inotrope/vasopressor	10	2 (0.7)	8 (10.0)	15.4 (3.3–70.8)	< 0.001
Need for ICU/HDU	42	32 (10.4)	10 (12.5)	1.2 (0.6–2.3)	0.595
Need for ventilator support	37	28 (9.1)	9 (11.3)	1.2 (0.6–2.5)	0.564
Cardiac arrest	9	2 (0.7)	7 (8.8)	13.4 (2.9–63.4)	< 0.001
24-h mortality	28	9 (2.9)	19 (23.8)	8.1 (3.8–17.2)	< 0.001

*CI* confidence interval

## Discussion

Emergency medicine is a new specialty in sub-Saharan Africa and having a fully functioning emergency department is an opportunity for quick and time-sensitive disease assessment, early resuscitation and stabilization of acutely ill patients with undifferentiated disease conditions. Worldwide, numerous studies have shown an increased level of serum lactate level is associated with amplified risks of developing serious adverse events [10, 25, 26], but whether that holds in our setting remains unclear. Patients presenting to our ED have a wide variety of diseases, many of which are rare in HIC’s, and often present much later in their illness.

In our study looking at serious adverse events for those emergency patients with lactate level obtained during their initial resuscitation, the mean lactate level was 3.2 ± 3.6 mmol/L. This mean value was low compared to the initial mean lactate level in a study done by Arnold et al. on ED patients with sepsis [27], also low compared to other studies done in similar populations of unselected disease patients in HIC’s [2, 28]. The possible reasons could be the diverse study population, inclusion criteria and differing study designs.

In our study, we found clinical characteristics like hypotension, tachycardia, tachypnoea, and hypoxia were statistically related to lactate level, even though some studies have shown lactate level to be higher in hemodynamically stable patients [13, 29]. Despite clinical characteristics being useful as a marker of patient severity, a study has shown these to have low sensitivity in predicting poor outcomes [27]. We, therefore, recommend the use of lactate level as a marker of poor prognosis in our set up as it is evidenced in our findings that a single lactate level value above 3.8 mmol/L would predict the critical state of a patient at high risk for mortality.

In this study, we found AUROC for the primary outcome of interest (24-h mortality) was 0.80, a *p* ≤ 0.001 (95%CI, 0.7–0.9); the best cut-off value for lactate level was >/= 3.8mmol/L with sensitivity and specificity of 64% and 85%, respectively. This is nearly similar to the cut-off of 4 mmol/L which has been found to have a significant association with SAEs in other settings of HIC’s [4, 25, 26]. The discrimination power for mortality was tolerable as per universal standards suggested by Metz CE (0.9–1 excellent, 0.8–0.9 good, 0.7–0.8 fair, 0.6–0.7 poor, 0.5–0.6 fails) [30]. In a systematic review on blood lactate level as a predictor of in-hospital mortality in patients admitted with acutely ill to hospitals lactate level of (2–4 mmol/L) lactate level had an area under the curve ranging around 0.7 to 0.80. Several studies have shown similar results in specific populations including sepsis, elderly, trauma, and cardiovascular disease [10, 13, 25].

The overall mortality in 24 h was 28 (7.3%), which is low compared to most of the studies done in similar populations in high-income countries. Again, this may be explained by the different settings and study designs noting that many of those studies outcomes were observed for more than 24 h duration [2, 13, 25, 28].

Based on the AUC for other SAE’s, lactate level performed well in predicting cardiac arrest and use of inotropes with a cutoff range of 3.5–3.6 mmol/L, but it did not perform well with the need for mechanical ventilator support or disposition to ICU, although this could be due to the various disease states and variety of patients enrolled, this finding might also be explained by resource constraints in our settings as admission to ICU/HDU or mechanical ventilation is often based on the ED circumstances and patient prognosis at MNH/MAMC will be factored in to conserve resources.

In this study, patients with abnormal potassium levels, acid-base balance disorders, and abnormal WBC were more likely to be associated with elevated lactate levels, therefore identifying a patient with high lactate levels could help to predict other common underlying abnormalities that could be easily managed in the ED. This can be useful in a resource-limited setting to avoid unnecessary testing in patients who are unlikely to have abnormalities.

Generally, our study findings are in keeping with recent literature on the predictive ability of lactate level as a prognostic biomarker of poor outcomes including mortality. Most literature has focused on disease-specific, e.g., sepsis, trauma [8, 13, 25] predictions with the risk of poor outcomes being reported to operate in a dose-dependent fashion [4]. In our study, there was a significant difference in SAE’s between those with lactate level above ^3^3.8 mmol/L compared to those with a lower level, despite the sensitivity being 64%. Overall, this would result in the exclusion of many patients. If the test was to be designed for screening purposes, especially in the ED, it might be desirable to choose a lower cut-off level with higher sensitivity to maximize capture; however, this may lead to inevitably over triaging, resulting in a greater challenge in terms of costs and sustainability especially in low-resource countries. In this study, lactate level was obtained using blood gas analyzers which are more expensive compared to a handheld lactate level meter which is less costly [31]. Since it was found that there was a predictive utility of lactate level in our settings, we recommend the procurement of a handheld lactate level meter with lactate level strips to increase the span of coverage and possibly reduce costs.

Several studies have shown the cutoff around 2–4 mmol/L and above 4 mmol/L predicts serious adverse outcomes and our findings are similar [5, 8, 25]. The relationship between illness severity and lactate level is not a new concept but to our knowledge, this was the first study assessing the POC serum lactate level concentration and SAEs in our population. The observed information may be useful for the development of protocolized management to maximize care to the ED population in our settings but also for further studies.

From this study, a cutoff of ^3^3.8 mmol/L and above has shown to have significant association with serious adverse events in our settings, this information may help to guide clinicians in decision-making and prediction of which patient might benefit with aggressive resuscitation, ICU or HDU, admissions and possibly close follow up; however, with development of protocolized management for lactate level clearance in our settings, all these may impact of care of our patient population.

## Limitations

Generalizability of our results may be limited as the study involved two tertiary centers that are 30 km apart, with the possibility of having similar populations characteristics, this may differ in other settings, particularly in limited-capacity ED found in another place of Tanzania or other LICs. On the other hand, the convenience sampling method was employed and only 387/2054 (18.8%) of the screened population was enrolled. However, we did not attempt to establish the clinical reasoning behind the decision to order lactate level, and this could be a source of bias as there were possibilities that there was a greater tendency to order lactate level when there was a greater concern of infection or critical illness. If lactate level were ordered routinely, this might result in a large number of false positives hence decreasing the discriminatory power of lactate level.

Some results of lactate level were not recorded in the patient’s records despite being ordered and the sample being sent, and the authors did not do follow up on reasons for the missing data hence probably we lost some eligible candidates to this study who may have contributed to the result in terms of deterioration or progress.

## Conclusions

A serum lactate level of 3.8 mmol/L predicted 24-h serious adverse events in high-acuity patients seen at our ED with no regard to any specific illness. Serum lactate level in the ED appears to be useful as a screening tool in a LIMC population to identify patients at risk of SAE; this information can be useful to bedside providers when incorporated into the appropriate clinical scenario and prove useful in clinical research aimed at optimizing and prioritizing care to our population.

## Recommendations

Lactate level appears to be a reasonable test in our settings for alerting providers of critically sick patients at risk of deterioration, but large studies are needed to possibly validate this findings as well as to see if those results are driven by specific diseases.

## Data Availability

The dataset supporting the conclusion of this article is available from the authors on request.

## Abbreviations

ED: Emergency Department
EMD: Emergency medicine department
ESI: Emergency Severity Index
HDU: High Dependency Unit
HIV: Human Immunodeficiency Virus
ICU: Intensive Care Unit
KCMC: Kilimanjaro Christian Medical Centre
LIC: Low-Income Countries
LMIC: Low- and Middle-Income Countries
MAMC: MUHAS Academic Medical Center
MNH: Muhimbili National Hospital
MUHAS: Muhimbili University of health and allied sciences
SAE: Serious Adverse Events/outcome
